# Associations of Hair Biomarkers with Height, Weight, and Body Mass Index in Preschool Children

**DOI:** 10.3390/children12091264

**Published:** 2025-09-20

**Authors:** Anjali Gupta, Cynthia R. Rovnaghi, Kanwaljeet J. S. Anand

**Affiliations:** 1 Child Wellness Lab, Maternal & Child Health Research Institute, Stanford University School of Medicine, Stanford, CA 94305, USA; anjalig@stanford.edu (A.G.); crovnagh@stanford.edu (C.R.R.); 2 Departments of Pediatrics and Anesthesiology, Stanford University School of Medicine, Stanford, CA 94305, USA

**Keywords:** early childhood, adversity, biomarkers, oxytocin, cortisol, overweight

## Abstract

**Background:** Adverse childhood experiences (ACEs) can lead to hypothalamic–pituitary–adrenal axis dysregulation, negatively impacting child growth and development. **Methods:** The Hair Biomarkers Study (HBS-I) evaluated healthy preschool children using parent-reported surveys, anthropometrics, and painlessly obtained scalp hair to measure hair cortisol (HCC; ng/mg) and oxytocin concentrations (HOC; ng/mg) via ELISA; log-transformed data were used in analyses. Height, weight, and BMI percentiles were based on CDC growth curves. Linear regressions examined associations of child and maternal hair biomarkers with percentiles, adjusting for sociodemographic factors. **Results:** The median age was 39.1 months (IQR 23.5–53.0, N = 1189). Higher child Ln-HCC was associated with an increased BMI percentile (0.027; 95%Cl: 0.013, 0.040), while higher child Ln-HOC was associated with a decreased BMI (−0.016; 95%Cl: −0.028, −0.005). Similar significant associations were observed between maternal biomarkers and child growth. In models mutually adjusted for mother and child hair biomarkers, maternal Ln-HCC was associated with an increased BMI percentile (0.036; 95%Cl: 0.003, 0.069), while maternal Ln-HOC was associated with a decreased BMI percentile (−0.034; 95%Cl: −0.052, −0.016). Maternal ACEs of 2–3 vs. 0–1 were associated with increased child weight (0.057; 95%Cl: 0.003, 0.112) and BMI percentiles (0.076; 95%Cl: 0.017, 0.135). **Conclusions:** We show that higher maternal and child HCC was associated with increased child BMI percentiles, while higher HOC was linked with decreased BMI percentiles. Hair biomarkers may reflect the effects of nurturing vs. adverse experiences during critical windows of growth and development in early childhood.

## 1. Introduction

There is growing evidence showing that early life adversity (ELA) and adverse childhood experiences (ACEs) can negatively impact developmental trajectories and health status among children [1,2,3,4,5,6]. Specifically, ELA is associated with being overweight in childhood and adolescence [7]. This is particularly concerning because excess body weight can lead to a range of chronic illnesses into adulthood, such as type 2 diabetes, cardiovascular disease, and hypertension [8,9,10]. Importantly, there are significant racial and socioeconomic disparities in the prevalence of ELA and obesity, with low-income and marginalized populations being disproportionately affected [11,12].

Many mechanisms have been proposed to explain the associations between ELA and excess body weight. The concept of allostatic load refers to the cumulative effects of fluctuating or heightened neural or neuroendocrine responses resulting from contextual or environmental stressors [13], causing chronic activation of the HPA axis [14]. Stress dysregulation can produce multisystem physiological dysregulation [15]. HPA axis activation leads to increased glucocorticoid production [14], which plays key roles in increasing appetite and visceral fat accumulation [16,17]. In acute stress, glucocorticoids regulate metabolism by mobilizing energy stores from glycogen and fats, and diverting energy away from anabolic processes, whereas chronic glucocorticoid excess promotes obesity, insulin resistance, and dyslipidemia [18].

Importantly, protective factors can moderate the association between ELA and poor health outcomes [19,20]. For example, children who reported being affectionate with their parents had decreased risk for health and developmental problems from exposure to ACEs, [21] and individuals who grew up with a trusted adult were less likely to report poor health despite exposures to childhood adversity [22]. Therefore, parental warmth and well-being are important protective factors for child development. Parental stress increases exposure to child ACEs, while reducing access to Positive Childhood Experiences (PCEs) and their protective effects [22,23]. Likewise, maternal traits like self-esteem can modulate maternal stress and produce improved parenting interactions that impact child HPA axis regulation [24,25]. Indeed, previous research has shown that higher parental stress is associated with child obesity [26].

While HPA axis dysregulation is thought to underlie the associations between ELA and obesity, the underlying physiological processes driving these outcomes remain unclear. Physiological biomarkers such as hair cortisol concentrations (HCC, measuring chronic stress [27]) and hair oxytocin concentrations (HOC, measuring social affiliation) reflect the stressful [28] vs. supportive [29] experiences of children. While cortisol represents stress-axis activation, oxytocin attenuates the HPA axis by (a) inhibiting neurons in the paraventricular nucleus (PVN) to reduce corticotropin-releasing hormone (CRH) release, (b) dampening amygdala and locus coeruleus inputs that stimulate the stress axis, while also having direct anxiolytic effects [30,31]. In addition to HPA axis inhibition, oxytocin also interacts with leptin signaling to reduce food intake, increase energy expenditure, increase lipolysis, enhance insulin sensitivity and thermogenesis, thus contributing to improved glucose homeostasis, burning of fat stores, and reduced body weight [32]. Cortisol and oxytocin values measured in saliva and serum are subject to diurnal variations and acute state reactivity [33,34], whereas painlessly obtained hair samples provide a chronic measure of these experiences [6]. Though several prior studies have related HCC with obesity and adiposity [35,36,37,38,39,40,41], a recent systematic review reported these findings to be inconclusive [42].

More rigorous evidence exploring HCC levels and child anthropometric measurements is warranted. Despite evidence of the interplay between stressful and supportive experiences on HPA axis dysregulation [19,20], no prior study has considered the role of HOC on child anthropometrics, and very few studies have explored parental biomarkers at all in relation to child growth and development, finding mixed results with HCC (i.e., positive, negative, and null associations) [35,43,44].

This study was designed to evaluate associations of child and maternal HCC and HOC, as objective biomarkers of stressful vs. supportive experiences, on height, weight, and body mass index (BMI) among preschool children. Given their opposing effects on the HPA axis, we hypothesized that higher child and maternal HCC would be associated with higher child BMI, while higher child and maternal HOC would be associated with lower BMI. We also evaluated the maternal self-reported measures of parenting stress, self-esteem, and ACEs in relation to their child’s anthropometric measurements. We hypothesized that greater parenting stress and ACEs would be associated with higher child BMI, while higher self-esteem would be associated with lower BMI. Potential covariates, such as dietary patterns, physical activity, or genetic predispositions, were not included in this study, which may influence the associations between stress hormones and body weight.

## 2. Methods

### 2.1. Hair Biomarkers Study—Phase I

Following IRB approval from Stanford University and written informed consent from parents, the cross-sectional Hair Biomarkers Study—Phase I (HBS-I) recruited families living in a geographically defined area (Santa Clara, San Mateo, and Alameda Counties) via local preschools, Head Start sites, faith-based groups, direct advertising at child-friendly locations, and Stanford-maintained parent listservs. The study evaluated healthy preschool children using parent-reported surveys, anthropometric measures, and painlessly obtained scalp hair (N = 1388). Parents also completed surveys about themselves, their family, and their child(ren), and mostly the mothers provided hair samples. Children with tinea capitis, alopecia areata, eczema, or other scalp conditions, those taking steroids or other prescription drugs, and those with chronic medical conditions, known developmental delay, or chemical exposures (e.g., dying, bleaching, chemical straightening, perming) to their hair in the 3 months prior to study entry were not eligible for participation.

### 2.2. Study Cohort

The current analysis was restricted to children with available data for sex, age, at least one hair biomarker (cortisol or oxytocin), and one anthropometric measurement (N = 1189). For analyses involving parent variables (i.e., self-reported surveys and parent hair biomarkers), maternal data were used. In the case of same-sex female couples, data were used from the parent designated “Parent 1” during data collection.

### 2.3. Hair Biomarker Assays

Child hair samples were obtained by painlessly trimming hair from a 1-cm^2^ scalp area near the posterior vertex (50–100 mg) using a Philips Norelco^®^ hair trimmer (Cambridge, MA, USA). Hair samples were processed following published methods [45]. Briefly, hair samples were cut to a powder consistency. Each sample underwent four successive extractions, alternating 1 mL of methanol (at 52 °C, 15 h) with 1 mL of acetone extraction (25 °C, 5 min). Supernatants from each extraction per sample were pooled for air evaporation at 4 °C. Dried residues were reconstituted in phosphate-buffered saline (PBS, pH 7.6) at 70 μL per 10 mg hair. HCC were determined using ALPCO cortisol ELISA kits (11-CRLHU-E01) (Salem, NH, USA) with developed plates read performed in a BioTeK plate using Gen 5 software (Winooski, VT, USA). HCC (ng/mg) and HOC (ng/mg) were measured in ELISA assays. Raw HCC and HOC values were log-transformed for use in analyses (i.e., Ln-HCC, Ln-HOC). Given that HOC and HCC may have opposing effects on the HPA axis [31], we calculated the molar ratio of HOC to HCC, multiplied it by 1000, and arbitrarily called it the Child Wellness Index.

### 2.4. Parent Surveys

As part of the HBS-I study, parents completed a series of self-reported surveys. Of relevance for the present analysis, parents completed the Parenting Stress Scale (PSS) [46], Rosenberg Self-Esteem Scale (RSE) [47], Felitti et al. [48] ACE instrument for parental ACEs, and ACE-Q instrument [49] for their child’s ACEs. The Parenting Stress Scale and Rosenberg Self-Esteem Scales were utilized as continuous variables in the analysis. Child ACEs were categorized as 1+ vs. 0, and parent ACEs were categorized as 0–1, 2–3, and 4+.

### 2.5. Outcomes

Research coordinators obtained child height and weight using standardized protocols. Anthropometric percentiles by age and sex were calculated via the Centers for Disease Control and Prevention (CDC) growth charts [50]. Height was calculated using charts titled, *stature-for-age, 2 to 20 years* (used for children 2 years or older) or *length-for-age, birth to 36 months* (used for children less than 2 years old). Weight was calculated using charts titled, *weight-for-age, 2 to 20 years* (children 2 years or older) or *weight-for-age, birth to 36 months* (children less than 2 years old). BMI percentiles by age and sex were calculated using charts titled, *BMI-for-age, 2 to 20 years* (children 2 years or older) or *weight-for-stature* (children less than 2 years old). BMI categories were calculated from their percentiles (underweight < 0.05 and overweight ≥ 0.85). Anthropometric percentiles and BMI categories were selected as the study outcomes because they are routinely measured and calculated during pediatric clinic visits.

### 2.6. Covariates

Covariates included parent-reported race/ethnicity (Hispanic, White, Asian, Mixed/Other), maternal marital status (married/living with partners, divorced/separated, single/widowed), child age (months, continuous), and socioeconomic status (SES). To assign SES, a four-level categorical variable was created based on annual household income and parental educational attainment.

### 2.7. Statistical Analysis

Participant demographics, anthropometric measurements, hair biomarkers, and self-reported surveys were described via median (interquartile range; IQR) for continuous variables and frequencies (%) for categorical variables. Linear regression models examined if child and maternal hair biomarkers (Ln-HOC and Ln-HCC) and a derived Child Wellness Index (molar ratio of child HOC/HCC values) were associated with the child’s height, weight, and BMI percentiles. Logistic regression models examined associations with child BMI category (underweight vs. normal; overweight vs. normal; overweight vs. underweight). First, each maternal and child biomarker was evaluated in separate regression models; then, maternal and child biomarkers were mutually adjusted for in the same model. We also examined associations of maternal PSS, RSE, ACEs, as well as child ACEs with child anthropometric percentiles and BMI categories in linear and logistic regression models, respectively. All models were run twice: (1) unadjusted; (2) adjusted for socioeconomic status, race/ethnicity, maternal marital status, and child age. All analyses were conducted in SAS version 9.4, with two-sided significance set at *p* < 0.05.

## 3. Results

### 3.1. Cohort Characteristics

Participant characteristics are reported in Table 1**.** The median age was 39.1 months (IQR 23.5–53.0), 56.3% were male, and 94.8% mothers were married or living with a partner (N = 1189). Most participants (51.5%) fell within the highest SES category, with only 6.9% in the lowest category. The median (IQR) height percentile was 0.58 (0.29, 0.84); weight was 0.50 (0.24, 0.77); and BMI was 0.49 (0.19, 0.80). Furthermore, 76% of children had zero ACEs, and 62.4% of mothers reported 0–1 ACEs.

### 3.2. Hair Biomarkers

Regression results for hair biomarker analyses are reported in Table 2. In unadjusted analyses, higher child Ln-HCC (−0.031; 95%Cl: −0.044, −0.019) and maternal Ln-HCC (−0.030; 95%Cl: −0.052, −0.010) were associated with a lower child height percentile. Each unit increase in the Child Wellness Index was associated with a 0.6 percentile increase in child height (0.006; 95%Cl: 0.0003, 0.011). Higher child Ln-HCC was associated with increased BMI percentile (0.029; 95%Cl: 0.016, 0.042), while higher child Ln-HOC was associated with decreased BMI percentile (−0.017; 95%Cl: −0.029, −0.005). Similar significant patterns were observed for maternal hair biomarkers. In models mutually adjusted for maternal and child biomarkers, maternal Ln-HOC was associated with a 2.7 percentile decrease in child BMI (−0.027; 95%Cl: −0.045, −0.009). Similar patterns were observed in logistic regression models by BMI category but reached statistical significance only in models comparing the underweight vs. normal weight and overweight vs. underweight children.

In analyses adjusted for sociodemographic characteristics, child growth patterns were consistent with the unadjusted analyses. Specifically, higher child Ln-HCC was associated with lower height percentile (−0.025; 95%Cl: −0.038, −0.012) and increased BMI percentile (0.027; 95%Cl: 0.013, 0.040), while higher child Ln-HOC was associated with decreased BMI percentile (−0.016; 95%Cl: −0.028, −0.005). Similar significant associations were observed between maternal biomarkers and child growth. In models mutually adjusted for mother and child hair biomarkers, maternal Ln-HCC was associated with a 3.6 percentile increase in BMI (0.036; 95%Cl: 0.003, 0.069), and maternal Ln-HOC was associated with a 3.4 percentile decrease in BMI (−0.034; 95%Cl: −0.052, −0.016).

### 3.3. Self-Reported Surveys

Regression results for hair biomarker analyses are reported in Table 3. In unadjusted analyses, maternal ACEs score of 2–3 vs. 0–1 was associated with a 7.2 percentile increase in child weight (0.072; 95%Cl: 0.017, 0.127) and a 9.2 percentile increase in child BMI (0.092; 95%Cl: 0.032, 0.151). These associations were attenuated but remained significant after adjusting for sociodemographic characteristics. Specifically, maternal ACEs score of 2–3 vs. 0–1 was associated with a 5.7 percentile increase in child weight (0.057; 95%Cl: 0.003, 0.112) and 7.6 percentile increase in child BMI (0.076; 95%Cl: 0.017, 0.135). No significant associations of child growth were observed with PSS, RSE, or child ACEs in both unadjusted and adjusted analyses.

## 4. Discussion

From the HBS-I cohort, we report significant associations of child and maternal hair biomarkers with child height, weight, and BMI percentiles. We found that higher child and maternal HCC were associated with decreased height and increased BMI percentiles, while higher child and maternal HOC were associated with increased height and decreased BMI percentiles. Mother HOC and HCC remained significant in models mutually adjusted for all four hair biomarkers, suggesting an effect beyond that explained by child biomarkers. We did not see any significant associations with the Child Wellness Index in adjusted models. Furthermore, maternal ACEs score of 2–3 vs. 0–1 was significantly associated with higher child weight and BMI percentiles.

Our results showing that higher child HCC was associated with increased BMI percentile is consistent with several other studies. Veldhorst et al. (The Netherlands) and Papafotiou et al. (Greece) showed that HCC is higher in obese children compared to normal-weight children [40,41]. While we did not see a significant association by BMI category, our results for the BMI percentile trend were in the same direction. Likewise, Sidote et al. (USA) and van der Valk et al. (The Netherlands) found that higher HCC was associated with higher BMI [51,52] and percent body fat [51]. Other studies in Australia, Greece and the USA, however, have reported no significant associations, which may in-part be related to sample size limitations, as these studies had significantly smaller sample sizes than ours [35,43,53]. Additionally, while we adjusted for several sociodemographic characteristics including maternal marital status, SES, child age, and race/ethnicity, other studies were more limited in the covariates they had included, though we did still see significant associations even in unadjusted models. Our results for HOC are unique given our ability to measure oxytocin in hair; however, they are consistent with data measuring oxytocin in the serum [54] as well as preclinical studies in murine models showing that deficiencies in oxytocin signaling and expression may lead to obesity [55].

We observed significant associations between maternal HOC and HCC and child anthropometric measurements. It is well-documented that high maternal stress places children at risk for obesity, while maternal warmth is a protective factor for growth and development [26,56,57]. However, few studies have documented associations between maternal hair biomarkers and child anthropometric measurements [35,43,44]. In contrast with our results, one study reported that preschoolers of mothers with higher HCC had significantly lower BMI z-score than those with mothers with lower HCC among a non-random sample of 35 mother-child dyads in the United States, [35] and another reported no significant associations [43]. Others have also reported that high maternal HCC was associated with a high fat mass index among offspring [44]. Although our sample size was greater than these conflicting studies, additional large studies may further clarify the associations between maternal stress and child growth/anthropometrics.

Despite the lack of prior studies evaluating associations between maternal hair oxytocin and child anthropometrics, our observed associations are consistent with similar published data. Oxytocin among parents is associated with higher synchrony and responsiveness in interactions with their children, and thus may promote HPA axis regulation in children [58]. Likewise, among children, oxytocin is associated with the development of secure attachment with their primary caregiver as well as prosocial behaviors [59,60]. Importantly, our data show that maternal HOC and HCC remained significant even after adjusting for child biomarkers. We studied preschool children, who are dependent on their parents for their basic needs and emotional inputs. Thus, parental biomarkers may be a stronger indicator of growth and development among children in this age range. Both these hormones and their signaling systems (receptors, etc.) also demonstrate high levels of heritability, which may further contribute to the observed associations between parent biomarkers and child outcomes [61,62].

Although HCC vs. HOC may reflect a child’s stressful [28] vs. supportive [29] experiences, we found no significant associations of their molar ratio with child anthropometrics in adjusted analyses. Oxytocin is believed to attenuate the stress axis by exerting an inhibitory effect on CRH release [30,31]. Indeed, exogeneous administration of oxytocin can reduce cortisol release in the blood stream, with stronger effects observed among clinical populations [63]. However, no prior work to our knowledge has evaluated the molar ratio of oxytocin to cortisol (measured in the same hair sample) to index the balance of stressful vs. supportive experiences that reflects overall child well-being. The Child Wellness Index used here is an unvalidated exploratory measure and is more likely to be associated with social, emotional, or mental health outcomes rather than the parameters of physical growth. Validation of this index is beyond the scope of the current analysis, but it warrants further investigation.

We found no significant associations of parent self-reported surveys (PSS, RSE) with child anthropometrics, consistent with the results of prior studies [43,64,65]. Timing may play an important role in this association, with data showing that postnatal maternal stress during the first year after birth had a positive longitudinal relationship with children’s BMI z-scores up to the age of five years [66]. Our cross-sectional study design was not able to confirm or refute this hypothesis. Although our results for RSE were not significant, maternal self-esteem may influence child BMI by affecting parenting interactions and the family environment, which have been related to child growth [26,56,57]. More importantly, we found that higher maternal ACEs (2–3 vs. 0–1) were associated with a 5.7 percentile increase in child weight and a 7.6 percentile increase in child BMI. This finding was consistent with our results for maternal HCC, showing that higher stress levels in mothers may often translate into increased risk of HPA axis dysregulation in their children [14,67].

## 5. Limitations

These findings must be interpreted with caution because of several limitations in this study. First, our study sample included predominantly high SES households, with over 50% of families falling within the highest SES category. Thus, our results may not be generalizable to lower SES populations, although past research confirms that ACEs are prevalent across different demographic groups, even among relatively wealthy populations [68,69]. Second, although some families enrolled multiple children in our study, we did not account for family level clustering, which may bias standard errors. However, this applies to less than 28% of families in our cohort, with 26% of families enrolling two children. Third, we acknowledge the potential of recall bias. While this may have affected our analyses utilizing parent-reported surveys, our main analyses focused on objective physiological biomarkers obtained from hair samples that would not be affected. Fourth, the cross-sectional nature of this study limits our ability to establish temporality or causality between hair biomarkers and child growth outcomes. Fifth, while hair biomarkers are validated as measures of chronic stress, their stability, contamination from sweat, and comparability across populations remain under debate, especially for oxytocin, where validation is less established. Sixth, we were unable to account for genetic predisposition, physical activity, and dietary factors, which may confound some of the observed associations. Important strengths of our study include our large sample size, and the availability of key sociodemographic covariates and parent psychosocial surveys.

## 6. Conclusions

We show that high maternal and child HCC was associated with increased child BMI, while higher HOC was associated with decreased BMI percentiles. This study adds significantly to the existing literature because our laboratory is uniquely capable of measuring hair oxytocin levels. Because objectively measured hair biomarkers can reflect the effects of nurturing vs. adverse experiences on early childhood growth and development, they should be considered as potentially useful measures for the screening of preverbal children during routine well-child visits. Early intervention, particularly among children at higher risk for ELA, may be beneficial.

## Figures and Tables

**Table 1:** Descriptive characteristics.

	No. Participants	N (%)
**Demographics**		
Age (mo.; median, IQR)	1189	39.09 (23.52, 52.95)
Sex	1189	
Male		669 (56.3)
Female		520 (43.7)
Self-Reported Race	1178	
Hispanic		134 (11.4)
White		422 (35.8)
Asian		327 (27.8)
Mixed/Other		295 (25.0)
SES Category	1178	
1		81 (6.9)
2		130 (11.0)
3		360 (30.6)
4		607 (51.5)
Mother’s Marital Status	1176	
Married/Living with Partner		1115 (94.8)
Divorced/Separated		17 (1.5)
Single/Widowed		44 (3.7)
**Anthropometric Measurements**		
Height Percentile (median, IQR)	1181	0.58 (0.29, 0.84)
Weight Percentile (median, IQR)	1187	0.50 (0.24, 0.77)
BMI Percentile (median, IQR)	1177	0.49 (0.19, 0.80)
Weight Category	1177	
Underweight (<5th percentile)		160 (13.6)
Normal Weight		794 (67.5)
Overweight (≥85th percentile)		223 (19.0)
**Hair Biomarkers**		
Child LN-HCC (median, IQR)	1183	1.53 (0.56, 2.30)
Child LN-HOC (median, IQR)	863	−7.47 (−8.90, −6.38)
Mother LN-HCC (median, IQR)	507	0.81 (−0.02, 1.54)
Mother LN-HOC (median, IQR)	428	−7.90 (−9.29, −6.79)
Child Wellness Index (median, IQR) ^1^	857	0.06 (0.01, 0.35)
**Survey Items**		
RSE Score (median, IQR)	819	25.0 (20.0, 28.0)
PSS Score (median, IQR)	824	38.0 (31.0, 44.0)
Child ACEs	909	
0		691 (76.0)
1+		218 (24.0)
Parental ACEs	736	
0–1		459 (62.4)
2–3		152 (20.7)
≥4		125 (17.0)

^1^ Child Wellness Index = (HOC molar/HCC molar) × 1000.

**Table 2:** Associations of hair biomarkers with anthropometric measurements.

**Height Percentile ^1^** Est. (95% CI)				
	Unadjusted	Sociodemographic-Adjusted ^a^	Mutually Adjusted ^b^	Sociodemographic/Mutually Adjusted ^a,b^
Child Oxytocin	0.009 (−0.003, 0.020)	0.005 (−0.006, 0.017)	−0.005 (−0.025, 0.015)	−0.009 (−0.029, 0.011)
Child Cortisol	−0.031 (−0.044, −0.019)	−0.025 (−0.038, −0.012)	−0.049 (−0.079, −0.019)	−0.032 (−0.064, 0.0005)
Mother Oxytocin	0.013 (−0.003, 0.028)	0.017 (0.001, 0.033)	−0.003 (−0.015, 0.021)	0.010 (−0.009, 0.028)
Mother Cortisol	−0.030 (−0.052, −0.010)	−0.027 (−0.048, −0.006)	−0.005 (−0.039, 0.029)	−0.016 (−0.049, 0.018)
Child Wellness Index	0.006 (0.0003, 0.011)	0.004 (−0.001, 0.010)		
**Weight Percentile ^1^** Est. (95% CI)				
	Unadjusted	Sociodemographic-Adjusted ^a^	Mutually Adjusted ^b^	Sociodemographic/Mutually Adjusted ^a,b^
Child Oxytocin	−0.007 (−0.017, 0.004)	−0.009 (−0.019, 0.002)	−0.007 (−0.025, 0.012)	−0.013 (−0.032, 0.005)
Child Cortisol	−0.004 (−0.016, 0.007)	0.003 (−0.009, 0.015)	−0.018 (−0.046, 0.010)	−0.009 (−0.038, 0.021)
Mother Oxytocin	−0.010 (−0.024, 0.005)	−0.008 (−0.022, 0.006)	−0.014 (−0.031, 0.003)	−0.015 (−0.031, 0.002)
Mother Cortisol	0.008 (−0.012, 0.027)	0.007 (−0.012, 0.027)	0.022 (−0.010, 0.053)	0.017 (−0.014, 0.048)
Child Wellness Index	0.003 (−0.001, 0.007)	0.002 (−0.002, 0.006)		
**BMI Percentile ^1^** Est. (95% CI)				
	Unadjusted	Sociodemographic-Adjusted ^a^	Mutually Adjusted ^b^	Sociodemographic/Mutually Adjusted ^a,b^
Child Oxytocin	−0.017 (−0.029, −0.005)	−0.016 (−0.028, −0.005)	−0.005 (−0.025, 0.015)	−0.008 (−0.028, 0.011)
Child Cortisol	0.029 (0.016, 0.042)	0.027 (0.013, 0.040)	0.028 (−0.003, 0.058)	0.012 (−0.019, 0.044)
Mother Oxytocin	−0.029 (−0.045, −0.013)	−0.031 (−0.047, −0.014)	−0.027 (−0.045, −0.009)	−0.034 (−0.052, −0.016)
Mother Cortisol	0.042 (0.021, 0.064)	0.036 (0.015, 0.058)	0.028 (−0.006, 0.062)	0.036 (0.003, 0.069)
Child Wellness Index	0.001 (−0.004, 0.007)	0.002 (−0.003, 0.007)		
**BMI Category: Overweight vs. Normal Weight ^2^** Odds Ratio (95% Cl)				
	Unadjusted	Sociodemographic-Adjusted ^a^	Mutually Adjusted ^b^	Sociodemographic/Mutually Adjusted ^a,b^
Child Oxytocin	0.96 (0.88, 1.06)	0.99 (0.90, 1.09)	1.00 (0.85, 1.18)	1.04 (0.86, 1.26)
Child Cortisol	1.10 (0.99, 1.22)	1.06 (0.94, 1.19)	1.15 (0.90, 1.48)	1.10 (0.82, 1.49)
Mother Oxytocin	0.95 (0.84, 1.08)	0.98 (0.85, 1.12)	0.95 (0.82, 1.10)	0.92 (0.78, 1.08)
Mother Cortisol	1.08 (0.90, 1.28)	1.04 (0.87, 1.25)	0.91 (0.69, 1.20)	0.90 (0.66, 1.22)
Child Wellness Index	1.03 (0.99, 1.07)	1.04 (0.99, 1.08)		
**BMI Category: Underweight vs. Normal Weight ^2^** Odds Ratio (95% Cl)				
	Unadjusted	Sociodemographic-Adjusted ^a^	Mutually Adjusted ^b^	Sociodemographic/Mutually Adjusted ^a,b^
Child Oxytocin	1.16 (1.04, 1.30)	1.18 (1.05, 1.33)	1.07 (0.88, 1.30)	1.14 (0.92, 1.41)
Child Cortisol	0.77 (0.69, 0.86)	0.77 (0.68, 0.87)	0.95 (0.72, 1.24)	0.95 (0.69, 1.31)
Mother Oxytocin	1.16 (1.01, 1.34)	1.21 (1.04, 1.40)	1.24 (1.03, 1.49)	1.28 (1.04, 1.57)
Mother Cortisol	0.72 (0.59, 0.86)	0.73 (0.60, 0.88)	0.61 (0.44, 0.85)	0.58 (0.40, 0.83)
Child Wellness Index	1.07 (0.99, 1.15)	1.07 (0.99, 1.15)		
**BMI Category: Overweight vs. Underweight ^2^** Odds Ratio (95% Cl)				
	Unadjusted	Sociodemographic-Adjusted ^a^	Mutually Adjusted ^b^	Sociodemographic/Mutually Adjusted ^a,b^
Child Oxytocin	0.83 (0.73, 0.95)	0.84 (0.73, 0.98)	0.94 (0.74, 1.19)	0.92 (0.70, 1.22)
Child Cortisol	1.40 (1.21, 1.61)	1.33 (1.14, 1.55)	1.38 (0.90, 2.10)	1.34 (0.77, 2.32)
Mother Oxytocin	0.80 (0.66, 0.97)	0.79 (0.64, 0.97)	0.74 (0.57, 0.97)	0.58 (0.40, 0.85)
Mother Cortisol	1.49 (1.17, 1.88)	1.38 (1.07, 1.78)	1.35 (0.88, 2.09)	1.56 (0.91, 2.68)
Child Wellness Index	1.01 (0.97, 1.05)	1.01 (0.97, 1.06)		

^1^ Linear regression models. ^2^ Logistic regression models. ^a^ Adjusted for socioeconomic status, race/ethnicity, mother marital status, child age. ^b^ Adjusted for all four biomarker variables in the same model.

**Table 3:** Associations of self-reported surveys with anthropometric measurements.

**Height Percentile** ^1^ Est. (95% CI)		
	Unadjusted	Sociodemographic-Adjusted ^a^
RSE Score	0.004 (−0.001, 0.008)	0.003 (−0.001, 0.008)
PSS Score	−0.002 (−0.004, 0.0005)	−0.002 (−0.004, 0.0006)
Child ACE (≥1 vs. 0)	0.030 (−0.018, 0.078)	0.032 (−0.019, 0.083)
Mother ACE		
0–1	Ref.	Ref.
2–3	−0.022 (−0.080, 0.036)	−0.023 (−0.082, 0.036)
≥4	0.045 (−0.018, 0.108)	0.052 (−0.014, 0.117)
**Weight Percentile** ^1^ Est. (95% CI)		
	Unadjusted	Sociodemographic-Adjusted ^a^
RSE Score	0.001 (−0.003, 0.005)	0.0002 (−0.004, 0.004)
PSS Score	0.0003 (−0.002, 0.003)	0.001 (−0.001, 0.003)
Child ACE (≥1 vs. 0)	0.0003 (−0.043, 0.049)	−0.019 (−0.067, 0.028)
Mother ACE		
0–1	Ref.	Ref.
2–3	0.072 (0.017, 0.127)	0.057 (0.003, 0.112)
≥4	0.047 (−0.012, 0.106)	0.023 (−0.037, 0.083)
**BMI Percentile** ^1^ Est. (95% CI)		
	Unadjusted	Sociodemographic-Adjusted ^a^
RSE Score	0.0004 (−0.004, 0.005)	−0.0004 (−0.005, 0.004)
PSS Score	0.001 (−0.001, 0.004)	0.002 (−0.001, 0.004)
Child ACE (≥1 vs. 0)	−0.029 (−0.078, 0.021)	−0.053 (−0.104, 0.001)
Mother ACE		
0–1	Ref.	Ref.
2–3	0.092 (0.032, 0.151)	0.076 (0.017, 0.135)
≥4	0.026 (−0.039, 0.090)	−0.005 (−0.070, 0.061)
**BMI Category: Overweight vs. Normal Weight** ^2^ Odds Ratio (95% Cl)		
	Unadjusted	Sociodemographic-Adjusted ^a^
RSE Score	0.99 (0.96, 1.04)	0.99 (0.96, 1.03)
PSS Score	0.99 (0.97, 1.01)	0.99 (0.97, 1.01)
Child ACE (≥1 vs. 0)	0.84 (0.57, 1.24)	0.80 (0.52, 1.23)
Mother ACE		
0–1	Ref.	Ref.
2–3	1.70 (1.12, 2.60)	1.64 (1.06, 2.54)
≥4	1.06 (0.65, 1.73)	0.88 (0.52, 1.48)
**BMI Category: Underweight vs. Normal Weight** ^2^ Odds Ratio (95% Cl)		
	Unadjusted	Sociodemographic-Adjusted ^a^
RSE Score	1.03 (0.99, 1.07)	1.04 (1.00, 1.08
PSS Score	0.98 (0.96, 1.00)	0.98 (0.95, 1.00)
Child ACE (≥1 vs. 0)	1.09 (0.65, 1.83)	1.25 (0.72, 2.16)
Mother ACE		
0–1	Ref.	Ref.
2–3	0.91 (0.46, 1.79)	1.04 (0.52, 2.07)
≥4	0.72 (0.34, 1.53)	0.93 (0.42, 2.03)
**BMI Category: Overweight vs. Underweight** ^2^ Odds Ratio (95% Cl)		
	Unadjusted	Sociodemographic-Adjusted ^a^
RSE Score	0.97 (0.92, 1.02)	0.96 (0.91, 1.01)
PSS Score	1.01 (0.99, 1.04)	1.02 (0.99, 1.05)
Child ACE (≥1 vs. 0)	0.77 (0.42, 1.40)	0.72 (0.37, 1.44)
Mother ACE		
0–1	Ref.	Ref.
2–3	1.88 (0.90, 3.90)	1.75 (0.78, 3.93)
≥4	1.47 (0.63, 3.40)	1.13 (0.44, 2.91)

^1^ Linear regression models. ^2^ Logistic regression models. ^a^ Adjusted for socioeconomic status, race/ethnicity, mother marital status, and child age.

## Data Availability

The data supporting this study will be available upon reasonable request to the corresponding author.
